# A Modern Approach to Brown-Séquard Syndrome: A Case Report

**DOI:** 10.7759/cureus.102036

**Published:** 2026-01-21

**Authors:** Lisle W Blackbourn, Sarah Khan, Jayishnu Srinivas, Johan Sosa De La Cruz, Swetha Vennavaram

**Affiliations:** 1 Neurology, University of Illinois College of Medicine Peoria, Peoria, USA; 2 Neurology, OSF Illinois Neurological Institute, Peoria, USA

**Keywords:** brown séquard, brown-séquard syndrome, ms, multiple sclerosis, myelopathy, neuromyelitis optica spectrum disorder, nmo, sarcoidosis, spinal cord infarct, spinal cord injury

## Abstract

In this case, we discuss an 82-year-old male patient with a combination of motor weakness and loss of proprioception on one side of the body, together with reduced pain and temperature sensation on the opposite side, which is characteristic of Brown-Séquard syndrome (BSS). This case highlights the differential diagnoses of those presenting with BSS as well as the diagnostic workup in the modern day for BSS. This case gives the reader the opportunity to go through tailored investigations and treatment for the various BBS differentials.

## Introduction

Brown-Séquard syndrome (BSS) is defined as the incomplete pattern of injury that manifests with damage to one half of the spinal cord, referred to as a hemisection. It has specific characteristic deficits of ipsilateral weakness and loss of vibration and proprioception sensation with contralateral loss of pain and temperature sensation. The consequent deficits arise from involvement of the corticospinal tract, dorsal column-medial lemniscus pathway, and spinothalamic tract [1].

Because any lesion that is confined to one half of the spinal cord may produce BSS, the full differential diagnosis for BSS includes both traumatic and nontraumatic etiologies. Traumatic causes, such as penetrating injuries, fractures, or dislocations, are the most common and should be considered in patients with a history of trauma [2,3]. However, nontraumatic causes are increasingly recognized and include structural, vascular, autoimmune, inflammatory, and neoplastic processes [1].

Structural causes of BSS include disc herniation, spinal stenosis, and epidural hematomas, which can compress the spinal cord and lead to hemisection [1,4]. Vascular causes, such as spinal cord infarction or hemorrhage, can also result in BSS, particularly if the anterior spinal artery is compromised [1]. Inflammatory and autoimmune conditions, such as multiple sclerosis (MS), neuromyelitis optica spectrum disorder (NMOSD), and sarcoidosis, are important considerations, especially in patients with a history of autoimmune disease or multifocal neurological symptoms [5]. Neoplastic causes, including primary spinal cord tumors and metastatic lesions, should also be considered, particularly in patients with systemic cancer or progressive neurological deficits [1,6,7].

While traumatic injury remains the most common cause of BSS, nontraumatic etiologies are increasingly recognized and include structural, vascular, inflammatory, and neoplastic processes. Each etiology presents with distinct clinical features, which can aid in diagnosis and management.

Here, we briefly present an 82-year-old male patient presenting with BSS symptoms to then go through the diagnostic work-up process, management of BSS, and further topics. Furthermore, our case highlights a newly diagnosed NMOSD case presenting at an advanced age.

## Case presentation

An 82-year-old man with a history of left cerebral meningiomas presented with a four-day history of progressive left lower extremity weakness and right lower extremity sensory changes. He also reported a chronic left facial droop, which had been present for approximately one year, and right abdominal burning pain that had started a few weeks prior.

On neurological exam, there was a mild left-sided facial droop. Motor strength testing showed significant weakness in the left lower extremity, with trace movement (1/5) in the hip flexors, extensors, knee flexion, and knee extension. Dorsiflexion and plantarflexion on the left were also severely impaired, with only slight movement against resistance (4/5). The right lower extremity and bilateral upper extremities had normal strength. Reflexes were hyperactive in the upper extremities but hyporeflexic in the left lower extremity, with normal reflexes on the right. Sensory testing revealed decreased pinprick and temperature sensation in the right lower extremity. Vibration sensation was decreased in both lower extremities, more pronounced on the left, and proprioception was absent in the left lower extremity. A sensory level was identified at T8/T9. The patient was alert and oriented, with no cognitive deficits, and speech was clear without evidence of dysarthria or aphasia.

**Figure 1:**
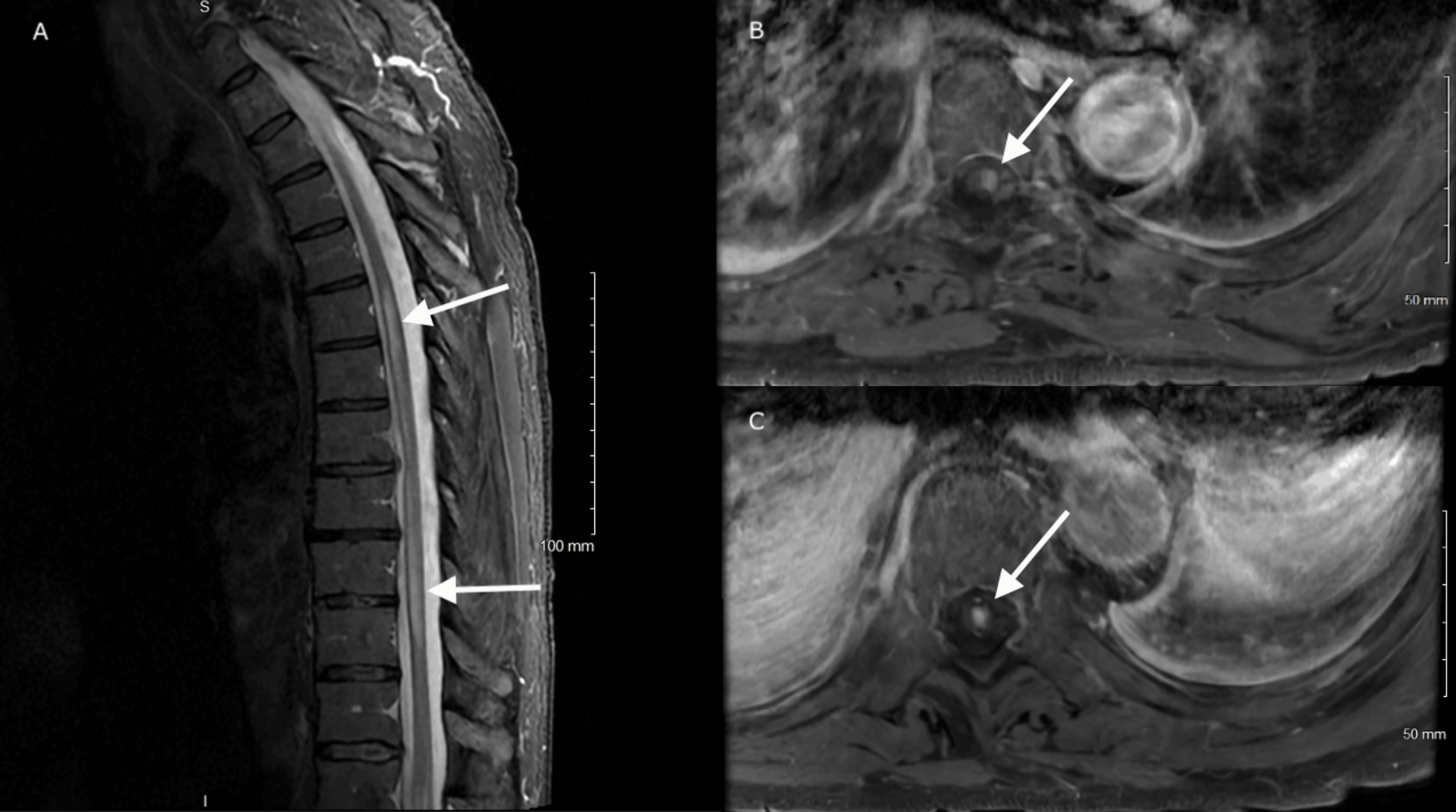
(A) Sagittal and (B and C) axial MRI T2 FLAIR hyperintensities in the thoracic spinal cord. FLAIR: fluid-attenuated inversion recovery

For our patient, imaging included a non-contrast CT head, which confirmed two left-sided meningiomas (2.7 cm and 2.5-2.6 cm) without acute intracranial abnormalities. MRI of the spine revealed a T6-T11 intramedullary T2 hyperintense lesion, thoracic degenerative spondylosis, and a T8-T9 disc herniation causing cord deformation, suggestive of possible disc herniation with anterior spinal artery compromise or an inflammatory/autoimmune process, as seen in Figure 1.

Vitamin and mineral testing was unremarkable. CSF studies showed elevated protein (72 mg/dL; normal range: 12-60 mg/dL), with all infectious testing unremarkable. AQP4-IgG testing was positive in both serum and CSF.

High-dose IV methylprednisolone was initiated, resulting in mild improvement in left lower extremity strength after two days. However, due to suboptimal improvement, plasmapheresis (PLEX) was started. The patient was also placed on vitamin supplementation (vitamin D, B12, and thiamine) until laboratory test results were within the normal range in case low levels were partially contributing to the presentation. Neurosurgery was consulted and recommended outpatient follow-up for the meningiomas. In follow-up in the outpatient clinic about six months later, the patient was noted to be able to walk with a walker with significant improvement in strength in the left leg (4/5 in all muscle groups). Sensory changes persisted as above but were reported as improved.

## Discussion

Differential diagnosis

The initial differential involved potential central nervous system pathologies, such as stroke, versus progression of his known meningiomas, versus a peripheral nerve issue. Most importantly, given his unilateral weakness and sensory changes, there was consideration given to spinal cord pathology. In particular, this patient’s combination of unilateral motor weakness and proprioceptive loss with associated contralateral pain and temperature sensory loss is characteristic of BSS [1].

As discussed earlier, any lesion that is confined to one half of the spinal cord may produce BSS. The full differential diagnosis for BSS includes both traumatic and nontraumatic etiologies. While traumatic injury remains the most common cause of BSS, nontraumatic etiologies are increasingly recognized and include structural, vascular, inflammatory, and neoplastic processes. Each etiology presents with distinct clinical features, which can aid in diagnosis and management.

Diagnostic work-up

The diagnostic work-up for BSS begins with a thorough clinical evaluation, including a detailed history and physical examination. The classic presentation of BSS includes ipsilateral motor weakness and loss of proprioception, along with contralateral loss of pain and temperature sensation, which localizes the lesion to one side of the spinal cord [1]. Imaging is the cornerstone of the diagnostic process, with magnetic resonance imaging (MRI) being the gold standard. MRI can identify structural abnormalities such as disc herniation, spinal cord compression, or intramedullary lesions, which are critical for determining the underlying cause [1,8]. In cases where MRI is inconclusive or unavailable, CT myelography may be used to assess spinal cord anatomy and identify compressive lesions.

CSF analysis is another essential component of the work-up, particularly in cases where an inflammatory or infectious etiology is suspected. Elevated protein levels, pleocytosis, or the presence of oligoclonal bands in the CSF may suggest an autoimmune or inflammatory process, such as MS or NMOSD [1,9,10]. Serological testing for autoimmune markers, such as aquaporin-4 antibodies (AQP4-IgG) and myelin oligodendrocyte glycoprotein antibodies (MOG-IgG), can further support the diagnosis of autoimmune-related BSS [9-11].

Laboratory studies, including vitamin B12, folate, and copper levels, as well as infectious serologies, are important to rule out metabolic or infectious causes of myelopathy [9-11]. In cases where neoplastic processes are suspected, imaging of the entire neuraxis and CSF cytology may be useful to identify primary or metastatic spinal cord tumors.

For our patient, remarkable workup included an MRI of the spine, which revealed a T6-T11 intramedullary T2 hyperintense lesion, thoracic degenerative spondylosis, and a T8-T9 disc herniation causing cord deformation, suggestive of possible disc herniation with anterior spinal artery compromise or an inflammatory/autoimmune process, as seen in Figure 1. This presented a diagnostic challenge given the coexisting structural lesions, and patients advance age for a new onset demyelinating process, thus prompting further workup. CSF studies showed elevated protein (72 mg/dL), with AQP4-IgG testing positive in both serum and CSF indicating NMOSD as diagnosis.

Management

Classical descriptions of traumatic BSS often involve penetrating injuries or fractures, such as those resulting from stabbings or falls, which lead to acute hemisection of the spinal cord [3]. These cases typically present with the hallmark features described above. Immediate surgical intervention has historically been emphasized in such cases to decompress the spinal cord and stabilize the spine, as delays in treatment were often associated with poor outcomes [1,12-14]. In cases of BSS secondary to structural causes, such as disc herniation or spinal stenosis, we often observe a more insidious onset of symptoms. Patients typically report chronic back pain or progressive neurological deficits, with imaging revealing compressive lesions at the level of the spinal cord. Surgical decompression has long been the mainstay of treatment for these cases [12,13].

Vascular causes of BSS, such as spinal cord infarction due to anterior spinal artery syndrome, have also been described [1,4,15]. These cases often present with sudden-onset paraplegia and sensory loss, particularly in patients with underlying vascular risk factors such as hypertension or atherosclerosis [1,13,16]. Historical management strategies have included anticoagulation and rehabilitation, with variable outcomes depending on the extent of spinal cord damage and the timeliness of intervention [13,14,16].

Inflammatory and autoimmune conditions, such as MS and NMOSD, have presented with subacute or relapsing-remitting symptoms [1,4,9,11]. Some case reports mention patients who develop BSS due to spinal cord lesions from autoimmune disease [5,10]. The diagnosis of autoimmune-related BSS relies on a combination of clinical, imaging, and laboratory findings. MRI typically shows focal or longitudinally extensive spinal cord lesions, while CSF analysis may reveal elevated protein levels, pleocytosis, or oligoclonal bands [1,5,9,10]. Serological testing for autoimmune markers, such as AQP4-IgG and MOG-IgG, can further support the diagnosis [9,11]. Treatment typically involves immunosuppressive therapy, such as high-dose corticosteroids, plasma exchange, or disease-modifying agents, depending on the underlying condition [9,11,12].

Neoplastic causes of BSS, including primary spinal cord tumors and metastatic lesions, have historically presented with progressive neurological deficits and systemic symptoms such as weight loss and fatigue. These are typically patients with intramedullary tumors, such as ependymomas or astrocytomas, who develop BSS due to spinal cord compression or infiltration. Surgical resection has historically been the primary treatment, with outcomes depending on the extent of tumor involvement and the timeliness of intervention [6-8].

For our patient, high-dose IV methylprednisolone was initiated, and later, PLEX was started for suspected NMOSD.

Further discussion

Recent research has significantly advanced the diagnosis and management of BSS, particularly in cases of nontraumatic etiology. Advances in imaging technology, such as diffusion tensor imaging (DTI) and functional MRI, have revolutionized the ability to detect subtle spinal cord lesions and assess spinal cord integrity. DTI, for instance, allows for the visualization of white matter tracts and can identify disruptions in spinal cord architecture that may not be apparent on conventional MRI [17,18]. This may be valuable in cases where traditional MRI findings are inconclusive or obscured by motion artifacts, as they provide a more detailed assessment of spinal cord pathology.

Additionally, the discovery of biomarkers has improved the ability to differentiate between inflammatory and non-inflammatory causes of myelopathy. Neurofilament light chain (NfL), a biomarker of axonal damage, has shown promise in identifying active inflammatory processes in the central nervous system [19-21]. Elevated levels of NfL in CSF are strongly associated with conditions such as MS and NMOSD, which can present as BSS [20]. This biomarker not only aids in diagnosis but also helps monitor disease activity and response to treatment, making it a valuable tool in the management of autoimmune-related BSS.

The role of immunotherapy in autoimmune-related BSS has been a major focus of recent research. PLEX has demonstrated efficacy in treating refractory cases of NMOSD and other autoimmune myelopathies, particularly in patients who do not respond adequately to high-dose corticosteroids [22,23]. PLEX works by removing pathogenic antibodies from the bloodstream, thereby reducing inflammation and preventing further damage to the spinal cord. Monoclonal antibodies, such as rituximab and tocilizumab, have also shown promise in targeting specific immune pathways involved in autoimmune myelopathies [24,25]. For example, rituximab, which targets CD20-positive B cells, has been effective in reducing relapse rates and the degree of disability in NMOSD patients, while tocilizumab, an interleukin-6 receptor antagonist, has shown potential in reducing relapse rates [24,25].

Ongoing clinical trials are exploring the use of stem cell therapy and neuroprotective agents to promote spinal cord repair and functional recovery in patients with BSS. The particular use of mesenchymal stem cells has shown potential in preclinical studies for their ability to modulate the immune response, reduce inflammation, and promote tissue regeneration [26]. Neuroprotective agents, such as riluzole and minocycline, are also being investigated for their ability to mitigate secondary injury and improve outcomes in spinal cord injury patients [27,28]. These therapies represent a promising frontier in the treatment of BSS, particularly for patients with nontraumatic etiologies who may not benefit from traditional surgical or medical interventions.

## Conclusions

While BSS remains a rare and understudied condition, there is still a diagnostic framework to identify the underlying cause. While traumatic injury remains the most common etiology, it is important to evaluate all nontraumatic causes. Structural, vascular, inflammatory, neoplastic, and autoimmune processes (MS, NMOSD) are all important etiologies of nontraumatic BSS and require prompt diagnosis and treatment to prevent long-term neurological deficits. A thorough clinical evaluation, combined with advanced imaging and laboratory studies, is essential for the accurate diagnosis and management of BSS. Recent research regarding these nontraumatic causes has provided significant insight into the pathophysiology and treatment of BSS, offering hope for improved outcomes in patients with this challenging condition.
